# Genetic diversity of the melanocortin-1 receptor in an admixed population of Rio de Janeiro: Structural and functional impacts of Cys35Tyr variant

**DOI:** 10.1371/journal.pone.0267286

**Published:** 2022-04-22

**Authors:** Vanessa Neitzke-Montinelli, Priscila da Silva Figueiredo Celestino Gomes, Pedro G. Pascutti, Rodrigo S. Moura-Neto, Rosane Silva

**Affiliations:** 1 Instituto de Biofísica Carlos Chagas Filho, Universidade Federal do Rio de Janeiro, Rio de Janeiro, Brazil; 2 Instituto de Biologia, Universidade Federal do Rio de Janeiro, Rio de Janeiro, Brazil; University of Chicago, UNITED STATES

## Abstract

The melanocortin-1 receptor (MC1R) is one of the key proteins involved in the regulation of melanin production and several polymorphisms have been associated with different phenotypes of skin and hair color in human and nonhuman species. Most of the knowledge is centered on more homogeneous populations and studies involving an admixed group of people should be encouraged due to the great importance of understanding the human color variation. This work evaluates the *MC1R* diversity and the possible impacts of *MC1R* variants in an admixed sample population of Rio de Janeiro, Brazil, which is a product of Native American, African, and European miscegenation. Sequencing of complete coding region and part of the 3´UTR of *MC1R* gene identified 31 variants including one insertion and three novel synonymous substitutions in sample population grouped according to skin, hair and eye pigmentation levels. In nonmetric multidimensional scaling analysis (NMDS), three main clusters were identified, in which the Brazilian dark skin group remained in the African cluster whereas the intermediate and the light skin color phenotype in the European one. None gathered with Asians since their immigration to Brazil was a recent event. *In silico* analyses demonstrated that Cys35Tyr, Ile155Thr and Pro256Ser, found in our population, have a negative effect on receptor function probably due to changes on the receptor structure. Notably, Cys35Tyr mutation could potentially impair agonist binding. Altogether, this work contributes to the understanding of the genetic background of color variation on an admixed population and gives insights into the damaging effects of MC1R variants.

## Introduction

Color variation is a complex trait orchestrated by elaborate signaling networks that are shaped by a set of genetic variants and epigenetic modifications. One of the most well studied genes governing the process of melanogenesis is the melanocortin-1 receptor (*MC1R*) that belongs to the family of G protein-coupled receptors (GPCRs) and encodes a protein with 317 amino acids composed of seven transmembrane domains (TM) with a high affinity for melanocyte stimulating hormone (MSH). *MC1R* is a highly polymorphic gene and it has been related to pigmentary as well as to non-pigmentary functions, including DNA repair (10.3390/genes12071093). MC1R plays a key role in skin protection against damaging ultraviolet radiation by regulating eumelanin production [1]. Preferentially present in melanocytes, the receptor activated by its agonists favors the synthesis of brown/black eumelanin instead of (yellow/red) pheomelanin, which predominates under condition of none or low signal transduction [2, 3]. The balance of eumelanin/pheomelanin production is a sophisticated mechanism to avoid the harmful consequences of ultraviolet (UV) exposure, not only in terms of DNA mutagenesis but mainly in maintaining folate integrity and vitamin D production [4]. For that reason, *MC1R* undergoes a directional selective pressure according to the geographic region reflected on the skin color: the more UV incidence, the darker the skin [4]. MC1R-defective individuals, because of their tendency to be undermelanized, they should accumulate more UV mutagenesis over time and would therefore be at higher risk for melanoma as a result [5, 6]. MC1R is overexpressed on the cell surface of most human melanomas making MC1R a valuable marker of these tumors. Although findings support association of genetic variants of MC1R of hair color and Parkinson’s disease (PD) risk [7] it is still controversial that variants of MC1R account for co-occurrence of PD and malignant melanoma [8].

*MC1R* is under strong evolutionary constraint in African origin since any underproduction of eumelanin appears to be deleterious [9]. As ancient humans migrated out of Africa towards low-UV regions in Europe or Asia, beneficial mutations have swept to fixation promoting convergent skin lightening [4, 10]. Most notably in European populations, many polymorphisms have been associated with fair skin phenotype, red hair color (RHC), presence of freckles as well as increasing predisposition of skin cancer [6, 11, 12]. The *MC1R* polymorphisms are best known for discriminating red hair from non-red hair phenotype due to mutations that impair the function of the receptor, disrupting the production of dark melanin [11]. The so-called strong alleles “R” for RHC have high penetrance for red hair and fair skin: Asp84Glu (rs1805006), Arg142His (rs11547464), Arg151Cys (rs1805007), Arg160Trp (rs1805008) and Asp294His (rs1805009). Some variants are predicted as damaging mutations such as Ser83Pro (rs34474212) and Pro256Ser (rs200215218) and also, variant for the loss-of-function Cys35Tyr (rs779504604) [13, 14].

Initially, studies on *MC1R* were based on individuals of unmixed origin who tend to have low intrapopulation skin color variation. However, few studies have been conducted in order to understand the genetic diversity and the phenotypic associations that comprise the intermediate levels between low and high melanin production, requiring further investigation in admixed populations [15–17]. Brazil is considered one of the most genetically diversified and highly admixed populations [18]. The process of colonization, undertaken firstly by Europeans with the contribution of Amerindians as native population, black Africans slaves and more recently Asians, has resulted in a broad range of color variations, more precisely in skin pigmentation [19]. In addition, the ethnic proportions and contributions of these parental populations (Amerindians, Africans and Europeans), observed in the Rio de Janeiro (RJ) population sample showed higher prevalence of European ancestry (around 60%) followed by African (25%) and native American (11%) [20], nevertheless, the distribution of skin color exhibits a wide range variation of phenotypes from low to high melanin content [21–23].

The present work approaches the genetic diversity of melanocortin-1 receptor in an admixed population with distinct color of skin, hair and eye. We sequenced the entire coding region and part of the 3´UTR of *MC1R* in a diverse population in terms of pigmentation levels and evaluated the phenotypic distribution related to skin color among the RJ population and those from the 1000 Genomes consortium [24], representing the parental contribution from colonization of Brazil to the present. Analysis based on population distribution, light and intermediate skin remains gathered to Europeans, despite the intermediate matrilineal ancestry of majority African origin. We also addressed the impact of distinct SNPs on the function and structure of the receptor using *in silico* methods. Variants associated with five point mutations (Cys35Tyr, Ile155Thr, Pro256Ser, Val156Leu and Phe196Leu), predicted as damaging mutations, were analyzed *in silico* to understand their structural effects and predict their functional roles on *MC1R*. Cys35Tyr is particularly significant due to the disruption of a critical disulfide bond and overall protein destabilization. Molecular dynamics simulations evidenced the mutation effect by structural conformational changes that potentially impair agonist binding. This work contributed to the understanding of the intermediate spectrum of color variation, and the functional implications of *MC1R* variants.

## Material and methods

### Population sample

134 unrelated individuals from Rio de Janeiro (Southeastern Brazil) with different pigmentation phenotypes of skin, hair and eyes (Table 1) were selected for *MC1R* genotyping. The population of study was selected non-randomly and the classification followed a qualitative analysis based on predetermined parameters of skin, hair and eye color. Collecting the biological material and the DNA extraction were described in a previous study [25]. The project was approved by the Ethics in Research Committee of Clementino Fraga Filho Hospital/UFRJ (CEP—MEMO—n.° 536/10). *MC1R* variants data from genetically unrelated individuals from parental populations such as Africans (GWD, LWK, MSL and YRI), Asians (CDX, CHB, CHS and JPT) and Europeans (FIN, GBR, IBS and TSI) were selected from the 1000 Genomes Project Consortium Phase 3 (for population keys see S1 Table) [26].

**Table 1:** Phenotypic frequency of admixed population from Rio de Janeiro.

Parameter	Individuals / n = 134
**Hair, *n (%)***	
Red	14(0.10)
Blond	25(0.19)
Light brown	16(0.12)
Dark brown	33(0.25)
Black	46(0.34)
**Skin, *n (%)***	
Light	75(0.56)
Intermediate	35(0.26)
Dark	24(0.18)
**Eye, *n (%)***	
Blue	21(0.16)
Green/Hazel	30(0.22)
Dark	83(0.62)
**Gender, *n (%)***	
Female	63(0.47)
Male	71(0.53)
**Age, *(years)***	
Mean (SD)	30.87(12.24)

### DNA genotyping and sequences analyses

Sanger sequencing was performed in all samples to encompass the human *MC1R* coding region. Amplification of the regions was performed with the GeneAmp High Fidelity enzyme of Applied Biosystems^™^ (30ng of DNA, 1x buffer, 1.5 mm MgCl_2_, 10% DMSO, 0.2mm dNTP, 1.5U of enzyme, 0.3μM of each primer—forward: 5*’-GGCAGCACCATGAACTAAGCAG*-3’ and reverse: 5’-*CAGGGTCACACAGGAACCAGAC-3*’ final volume of 50μl—cycling: 94°C 2min; 30x (94°C 20s, 63°C 20s, 72°C 1min) 72°C 7min. The amplified product was purified by PCR Cleanup kit (AxygenTM) and sequenced using Big Dye Terminator (Thermo Fisher, CA). The products were run in Applied Biosystems^™^ ABI 3130xl, using six primers designed by our group (5*’-GAAGAACTGTGGGGACCTGGA*-3’, 5*’-CAGGAAGCAGAGGCTGGACAG*-3’, 5*’-ATGTACTGCTTCATCTGCTGC*-3’, 5*’-CAGGATGGTGAGGGTGACAGC*-3’, 5*’-TCCTGGCTATGCTGGTGCTCA*-3’, 5’-*ACACAATATCACCACCTCCCTCT*-3’) which covered the intronless *MC1R* coding region and part of 3’UTR at least twice. Sequences from both strands were aligned with the SNP-contained GenBank consensus sequence format from *Homo sapiens* chromosome 16, reference assembly, complete sequence (GenBank version NC_000016.8 GI:51511732), using the blast tool from Geneious Pro 4.7 software (Biomatters). The region of HVI of mitochondrial DNA was sequenced based on the protocol described by another group [10] and the classification of haplogroups was achieved through the command line version of Haplogrep v.2.2.6 [27]. The correspondent geographic region for haplogroups was determined through the MITOMAP database [28]. For the parental populations analysis, all the polymorphisms from *MC1R* gene (89985667–89986632bp) were extracted from The 1000 Genomes database (ftp.1000genomes.ebi.ac.uk/vol1/ftp/release/20130502/) using the VCFtools v.0.1.15 [29] and the variant annotations were obtained by SnpEff v.4.5 package [30].

### Statistical analysis

The allele frequencies and the Hardy-Weinberg equilibrium (HWE) were calculated using Adegenet v. 2.1.3 and Pegas v.0.13 packages [31, 32], respectively. The phylogenetic tree was obtained by applying the neighbor joining method (NJ function) using ape v.5.3 package [33]. To evaluate the clusters of skin color individuals from RJ and the 1000 Genomes populations, the nonmetric multidimensional scaling (NMDS) and its respective stress plot were conducted with the vegan v.2.5.6 package based on two dimensions (K), kulczynski distance and 25 as a maximum number of random starts. The phylogenetic tree and all the multidimensional scaling plots were based on the *Fst* genetic distance that were performed through Nei’s pairwise *Fst* calculation using the pairwise.neifst function and the respective confidence interval, boot.ppfst () function, both from hierfstat v.0.5.7 package [34]. All the analyzes above were performed using the R-studio v.1.3.1056. The R packages ggplot and ggtree were used to generate the graphs and trees, respectively.

### *MC1R* mutational analysis

Mutational analyses were performed using two criteria: i. Based on the protein sequence; ii. Based on the protein three-dimensional (3D) structure. For the sequence-based predictions, the following disease-association predictors were used: PolyPhen [35], PON-P2 [36] and Meta-SNP [37], based on the Uniprot ID Q01726. For the structure-based predictions, the following predictors were used: DUET [38] and DynaMut [39]. DUET includes a consensus score and the individual scores for mCSM and SDM predictors.

### *MC1R* model construction

The *MC1R* sequence was retrieved from the UniProt database (ID: Q01726) [40]. Template search with BLAST and HHBlits against the Protein Data Bank (PDB) [41] were performed using the SWISS-MODEL server [42]. The best template was the crystal structure of the human melanocortin receptor 4 (MC4R) (PDB ID: 6w25) with sequence identity of 50.18% and alignment coverage of 0.87. Since this crystal did not cover a small region (residues 29–38) of the target protein containing a mutation site on position 35, we selected an additional template, the muscarinic acetylcholine receptor M2 (PDB ID: 5zk8), with 20.21% sequence identity but a slightly higher coverage value of 0.89. Multiple template modelling of the wild-type (WT) *MC1R* was performed using MODELLER [43] version 9.23, using standard parameters. The best model was selected based on DOPE score [44]. Modelling of the five selected mutants was also performed through MODELLER, using as template the generated WT model.

### Molecular dynamics simulations

The effect of Cys35Tyr mutation was simulated under three scenarios:

1. C35: WT in absence of the disulfide Cys35-Cys275 bond;
2. C35-C275: WT with the disulfide Cys35-Cys275 bond;
3. Cys35Tyr mutant

A disulfide bridge was introduced between cysteine residues 35 and 275 using MODELLER. All systems were submitted to a molecular dynamics (MD) simulation for refinement using AMBER 18 [45]. The protein was solvated on a cubic box with a 1.2 nm distance to the faces under periodic boundary conditions (PBC) using the TIP3P water model [46] and minimized in three stages, for 500 steps each, using steepest descent followed by conjugate gradient, restraining the movement for all the heavy atoms including ions, heavy atoms excluding ions, heavy atoms excluding ions and the backbone, respectively. After the minimizing process, system equilibration was performed on the solvent/ions in three stages: (i) gradually heating the system from 0° to 100° K for 50ps under the NVT ensemble with weak position restraints for the protein heavy atoms, using a weight of 2 kcal/mol−Å2; (ii) heating from 100° to 298.15° K for 50ps under the NPT ensemble with weak position restraints for the protein heavy atoms, using a weight of 2 kcal/mol−Å2; (iii) MD under the NPT ensemble at constant temperature of 298.15° K for 500ps with very weak position restraints for the protein heavy atoms, using a weight of 1 kcal/mol−Å2. Two replicas for the production run were carried out for 100ns under the NPT ensemble, for each scenario. All production runs were visualized and checked for system convergence prior to analysis.

### *MC1R* conformational dynamics

The volume of the hormone-binding pocket of MC1R was evaluated over the MD simulations for the three scenarios using MDPocket [47]. The two MD replicas, for each scenario, were concatenated for the calculation. Root Mean Square fluctuations (RMSF) and cluster analyses were calculated using the *cpptraj* module available with AmberTools 18 [48] over both replicas, for each system. The k-means algorithm was used to generate 10 clusters considering only the protein backbone with distance metric parameters: rms; sieve 10. One representative structure was generated for each cluster. The representative conformation displayed corresponds to the most populated cluster.

## Results

### *MC1R* is highly polymorphic in an admixed population

Assessing the genetic diversity of the intronless *MC1R* gene, we observed that 75% of Rio de Janeiro (RJ) sample population presented at least one polymorphism. These 31 variants encompasses 14 synonymous mutations, 15 non-synonymous, one indel variation and one SNP at 3’UTR. From those, 28 were already described polymorphisms and three were novel synonymous variants in heterozygous state (Leu11Leu, Tyr143Tyr and Ala181Ala; Table 2). Interestingly, the novel polymorphism found in humans (c.429 C>T, p.143 Tyr>Tyr) was also observed in two different breeds of sheep [49, 50]. It is probable that the emergence of this variation shared between humans and sheep occurred as independent events.

**Table 2:** Minor allele frequency (MAF) of *MC1R* polymorphisms from RJ population.

SNP ID	Nt / Aa change	MAF (n)*	Skin			Hair					Eyes		
			Light	Intermed	Dark	Red	Light	L. brown	D. brown	Black	Blue	Green/Hazel	Dark
Novel SNP	c.31C>T / Leu11Leu	0.004 (1)	-	-	0.021	-	-	-	-	0.011	-	-	0.006
rs779504604	c.104G>A / Cys35Tyr	0.007 (2)	0.013	-	-	0.071	-	-	-	-	-	0.017	0.006
rs1805005	c.178G>T / Val60Leu	0.104 (28)	0.133	0.100	0.021	0.107	0.100	0.250	0.091	0.065	0.190	0.150	0.066
rs34474212	c.247T>C / Ser83Pro	0.011 (3)	0.020	-	-	0.071	0.020	-	-	-	-	0.017	0.012
rs1805006	c.252C>A / Asp84Glu	0.015 (4)	0.027	-	-	0.036	0.060	-	-	-	-	0.067	-
rs2228479	c.274G>A / Val92Met	0.019 (5)	0.020	-	0.042	-	-	0.031	0.030	0.022	0.024	-	0.024
rs780284801	c.288C>T / Ala96Ala	0.004 (1)	-	0.014	-	-	-	-	0.015	-	-	-	0.006
rs140650544	c.309C>T / Ala103Ala	0.004 (1)	-	0.014	-	-	-	-	0.015	-	-	-	0.006
rs3212364	c.318G>A / Leu106Leu	0.007 (2)	-	-	0.042	-	-	-	0.015	0.011	-	-	0.012
rs201429598	c.399C>T / Cys133Cys	0.004 (1)	0.007	-	-	-	-	0.031	-	-	-	-	0.006
rs11547464	c.425G>A / Arg142His	0.011 (3)	0.013	0.014	-	0.036	-	-	0.030	-	-	0.033	0.006
Novel SNP	c.429C>T / Tyr143Tyr	0.004 (1)	0.007	-	-	-	-	-	0.015	-	-	-	0.006
rs1805007	c.451C>T / Arg151Cys	0.041 (11)	0.073	-	-	0.214	0.060	-	0.030	-	0.024	0.050	0.042
rs201827012	c.453.C>G / Arg151Arg	0.004 (1)	0.007	-	-	-	-	0.031	-	-	-	-	0.006
rs1110400	c.464T>C / Ile155Thr	0.007 (2)	0.013	-	-	-	0.040	-	-	-	-	0.017	0.006
rs3212365	c.466G>C / Val156Leu	0.004 (1)	-	-	0.021	-	-	-	-	0.011	-	-	0.006
rs1805008	c.478C>T / Arg160Trp	0.015 (4)	0.027	-	-	0.036	0.040	-	0.015	-	0.024	-	0.018
rs885479	c.488G>A / Arg163Gln	0.056 (15)	0.047	0.086	0.042	-	0.040	0.125	0.045	0.065	0.071	0.050	0.054
rs34612847	c.504C>T / Ile168Ile	0.004 (1)	-	0.014	-	-	-	-	0.015	-	-	-	0.006
rs555179612	c.537_538insC / Frameshift	0.004 (1)	0.007	-	-	0.036	-	-	-	-	-	-	0.006
Novel SNP	c.543C>T /Ala181Ala	0.007 (2)	0.013	-	-	-	0.040	-	-	-	-	0.033	-
rs370040645	c.546C>T / Tyr182Tyr	0.004 (1)	0.007	-	-	0.036	-	-	-	-	-	0.017	-
rs3212366	c.586T>C / Phe196Leu	0.015 (4)	-	0.029	0.042	-	-	-	-	0.043	-	-	0.024
rs146544450	c.699G>A / Gln233Gln	0.004 (1)	-	-	0.021	-	-	-	-	0.011	-	-	0.006
rs200215218	c.766C>T / Pro256Ser	0.004 (1)	0.007	-	-	0.036	-	-	-	-	-	0.017	-
rs375813196	c.819C>T / Cys273Cys	0.004 (1)	-	-	0.021	-	-	-	-	0.011	-	-	0.006
rs1805009	c.880G>C / Asp294His	0.030 (8)	0.053	-	-	0.286	-	-	-	-	0.024	0.067	0.018
rs3212367	c.900C>T / Phe300Phe	0.019 (5)	-	0.043	0.042	-	-	-	0.015	0.043	-	-	0.030
rs375127718	c.923C>T / Thr308Met	0.004 (1)	0.007	-	-	-	-	0.031	-	-	-	0.017	-
rs2228478	c.942A>G / Thr314Thr	0.164 (44)	0.080	0.143	0.458	-	0.080	0.063	0.167	0.293	0.095	0.083	0.211
rs3212368	c.*12G>A / 3´UTR	0.045 (12)	0.007	0.029	0.188	-	0.020	-	0.015	0.109	-	0.017	0.066

The table refers to the minor allele frequency (MAF) of each polymorphism in a total population and in the skin, hair and eye color subgroups. N: Number of alleles in a population; hyphen: No minor allele was observed.

The analysis of the *MC1R* variants showed that all the polymorphisms are in HWE with the exception of Val60Leu (rs1805005). The Val60Leu, Arg163Gln, Thr314Thr and rs3212368 from 3’UTR were the SNVs with the highest frequencies in RJ population with minor allele frequency (MAF) equal to or above 5% (Table 2). Despite the purifying selection, nonsynonymous variants were already detected in African populations. In our study, four individuals with high levels of melanin carry the variant Phe196Leu (rs3212366), which is located on the fifth transmembrane domain, previously identified in the African sub-Saharan population [51]. In addition, the variant Val156Leu (rs3212365) which is so far detected only in the African population according to the 1000 Genome Project (S2 Table), was identified in one black individual in our sample.

Regarding the so-called strong alleles “R” for RHC that have high penetrance for red hair and fair skin, we detected the Asp84Glu (rs1805006), Arg142His (rs11547464), Arg151Cys (rs1805007), Arg160Trp (rs1805008) and Asp294His (rs1805009). In the same group, we found an insertion of a cytosine at nucleotide position 537 (rs555179612) of the *MC1R* ORF, encoding a truncated protein 237 amino acids in length in the transmembrane 4 domain region. In our sample population, minor allele frequency (MAF) of two variants previously predicted as damaging mutations, Ser83Pro (rs34474212) and Pro256Ser (rs200215218), also the loss-of-function SNP Cys35Tyr (rs779504604), were present in 1.1%, 0.4% and 0.7% respectively (Table 2). Cys35Tyr was detected in two red-haired individuals in heterozygous condition. One person also carried a second variant, Ser83Ser/Pro, previously associated with changes in the structure and function of the receptor [14], and the other individual exhibited Cys35Tyr as the unique variant detected in the entire sequence of the *MC1R* coding region.

### Intermediate skin color group from RJ is genetically closed to European population based on *MC1R* variation

**Fig 1:**
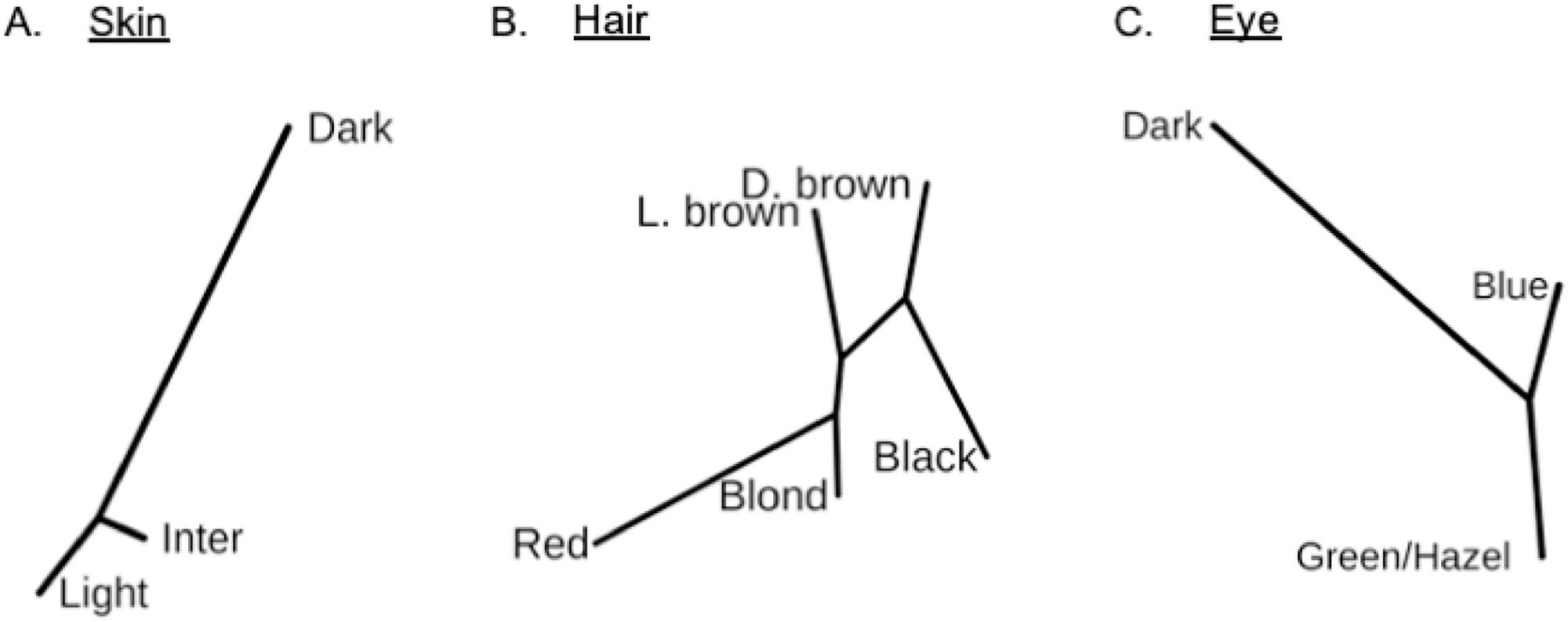
Color phenotype profile based on *MC1R* variation in an admixed population. Phylogenetic tree based on neighbor-joining method using the F*st* distance of different groups of (**A**) skin, (**B**) hair and (**C**) eye colors.

To understand the relationship among color phenotypes based on the overall *MC1R* variation in miscegenated individuals, we assessed the distribution pattern of the different phenotype within our population from low to high melanin content for skin and hair color phenotypes using neighbor-joining phylogenetic trees with *Fst*-pairwise genetic distances (Fig 1A and 1B and S3 and S4 Tables).

The red-haired group was positioned relatively distant from other phenotypes with a large *Fst* distance as well as the lighter-colored eyes from the dark ones (Fig 1C and S4 Table). An interesting finding was observed relating to the skin color variation shown in Fig 1A. The intermediate skin color group is closer to the light skin color group than the dark one, indicating a genetic distance similarity between both groups.

**Fig 2:**
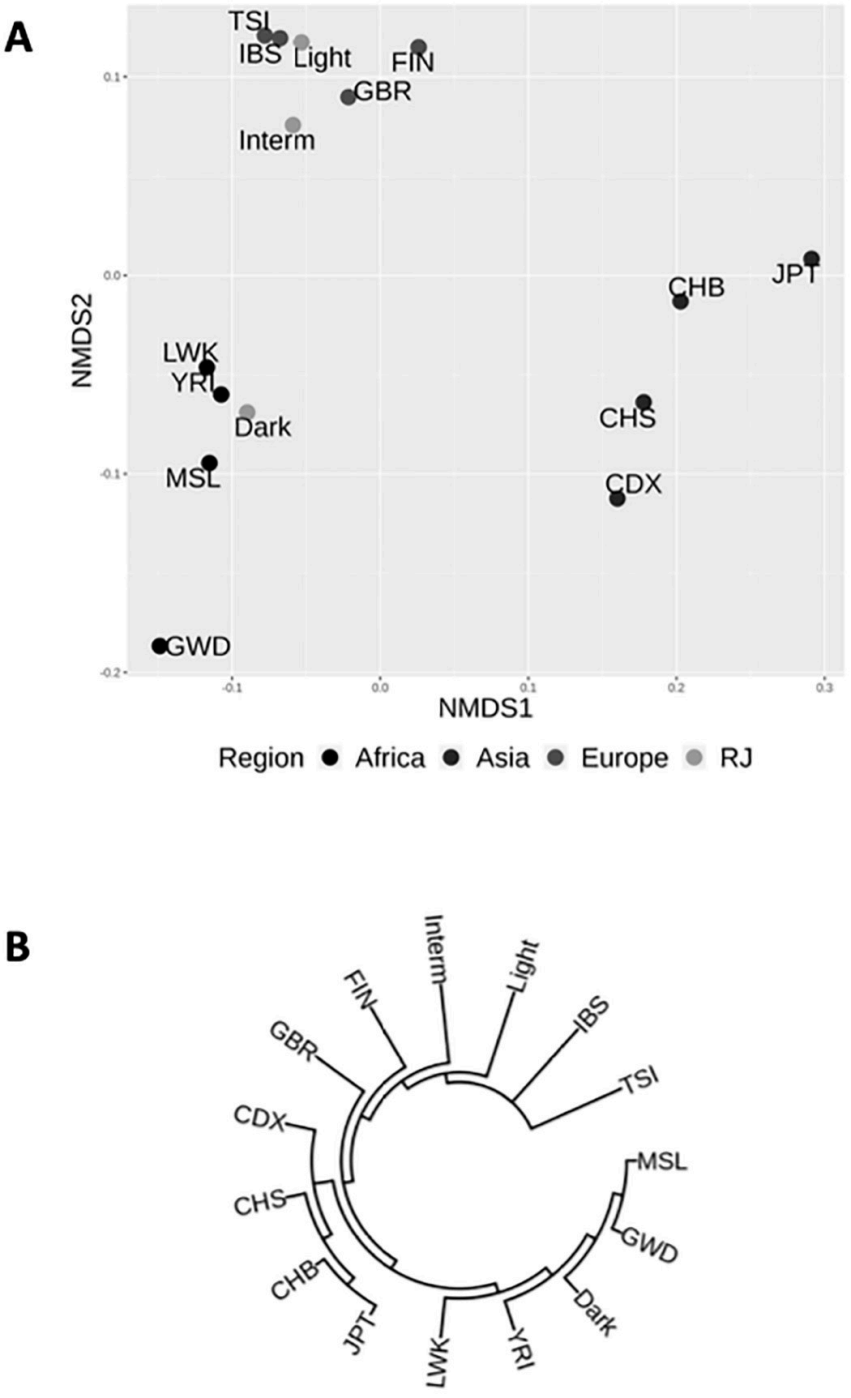
Distribution of skin color phenotype from RJ compared to parental populations from the 1000 Genomes project. (**A**) The nonmetric Multidimensional Scaling (NMDS) analysis was performed based on Fst analysis of the light, intermediate and dark skin colors among 12 populations in Africa (GWD, LWK, MSL, YRI), Asia (CDX, CHB, CHS, JPT) and Europe (FIN, GBR, IBS, TSI) obtained from the 1000 Genomes Consortium. (**B**) Phylogenetic tree based on the *Fst* genetic distance. **Africa** (GWD-Gambian in Western Divisions; LWK-Luhya in Webuye, Kenya; MSL-Mende in Sierra Leone; YRI-Yoruba in Ibadan, Nigeria); **Asia** (CDX-Chinese Dai in Xishuangbanna, China; CHB-Han Chinese in Beijing, China; CHS-Southern Han Chinese); JPT-Japanese in Tokyo, Japan); **Europe** (GBR-British in England and Scotland; FIN-Finnish in Finland; IBS-Iberian Population in Spain; TSI-Toscani in Italia).

Considering the variation of the Brazilian skin color is due to the miscegenation of black Africans, medium-tone Native American and light skin Europeans, it is not known whether the similar genetic distance between the light and intermediate skin phenotypes is a result of a high contribution of European background in the intermediate group or whether the light skin color is mixed enough that it differs from white Europeans. To comprehend this issue, we assessed the distribution of the skin color phenotype samples compared to African, Asian and European populations from the 1000 Genomes Project (S2 Table) by performing a nonmetric multidimensional scaling (NMDS) analysis based on the pairwise *Fst* genetic distance (S3 Table). Running with k = 2 dimensions (R^2^: 0.998, stress: 0.0206), we observed three distinct clusters corresponding to the populations of Africa (GWD, LWK, MSL and YRI), Asia (CDX, CHB, CHS and JPT) and Europe (FIN, GBR, IBS and TSI) (Fig 2A).

Moreover, the elements on dimension 2 of the plot were able to distinguish the melanin content in the skin, in which the pigmentation levels decreased as the values increased. This conception in the NMDS data is strengthened by the distribution of Asian cluster. This distribution is corroborated by previous reports that showed the southern Asians with lower skin reflectance compared to the northern ones from Beijing and Japanese [52]. Related to our population, the admixed light skin individuals gathered with Europeans (Fig 2A and 2B), in particular Mediterraneans, differently from the dark skin individuals who are grouped with Africans, closer to Kenyans and Nigerians. However, the intermediate skin phenotype was close to the light skin, since the *Fst* distance is small between both groups compared to dark skin color (Figs 1 and 2 and S3 Table), suggesting that coding region sequence of *MC1R* would discriminate, more properly, dark from non-dark skin color in an admixed population from RJ.

**Fig 3:**
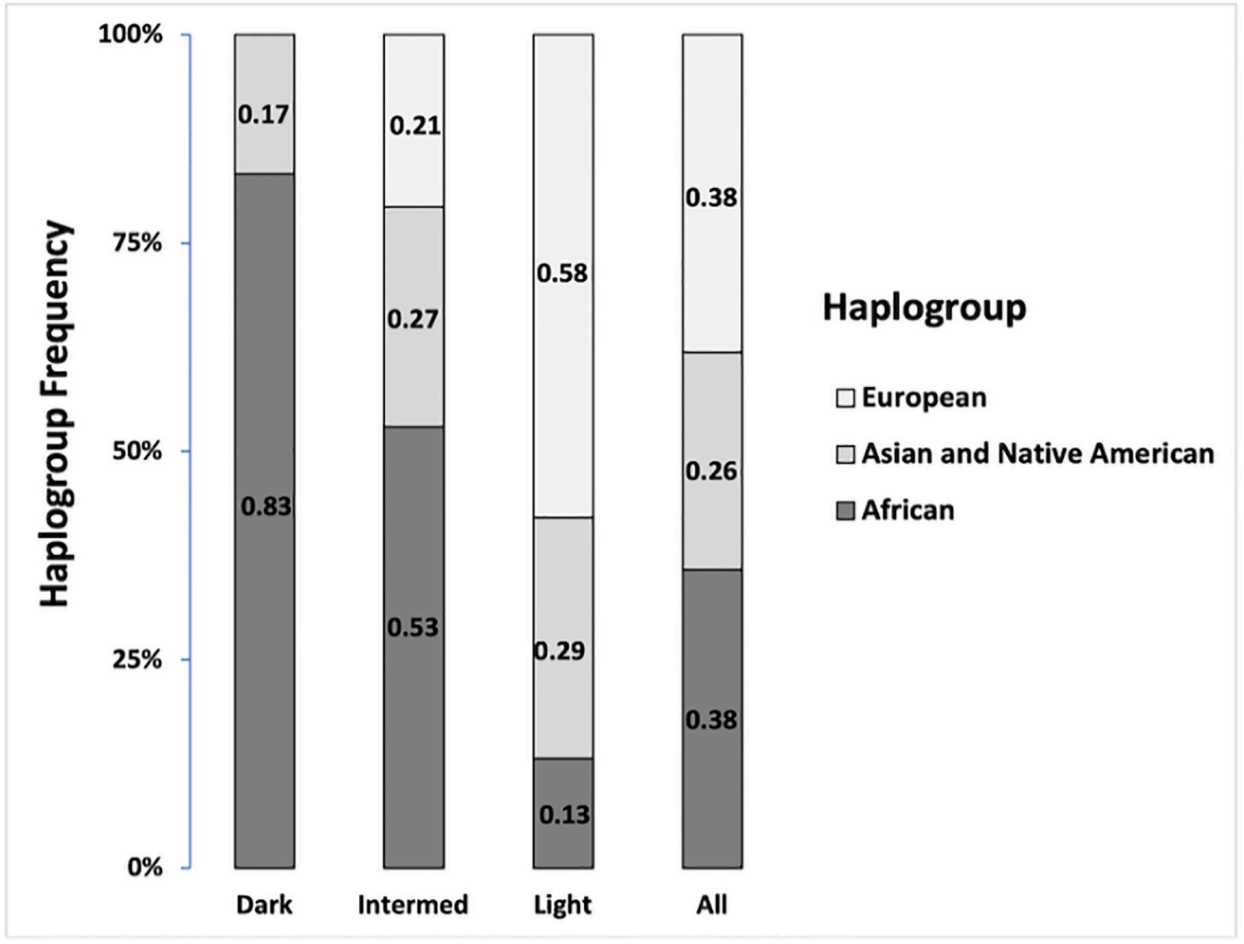
Distribution of matrilineal lineages among individuals of skin color variation in the population of RJ. Haplogroups based on the HVS-I region of Mitochondrial DNA were classified using Haplogrep software. The numbers correspond to the frequency of African, Asian, Native American and European haplogroups within color phenotypes: Dark, intermediate and light. All represents the frequency distribution of matrilineal lineages of all skin color variation in our sample.

To support the above data, we analyzed the matrilineal genetic ancestry of the 134 individuals. We observed that the mitochondrial DNA haplogroups exhibit a distinct distribution among skin color phenotype, wherein the light and dark skin groups showed a dominant proportion of European and African ancestry, respectively (Fig 3 and S5 Table).

Interestingly, the intermediate phenotype displayed majority contribution of African mtDNA (53%) and similar proportion between Native American and European, 26.5% and 20.6%, respectively, not supporting the fact that intermediate skin color clusters near to the light color phenotype through matrilineal genetic ancestry analysis.

### Nonsynonymous polymorphisms are predicted to impact negatively on *MC1R* function

We evaluated the amino acid changes that potentially interfere with the function of the MC1R protein receptor. Since many nonsynonymous polymorphisms found in our sample were extensively studied regarding their effects on melanin production, we decided to focus on five nonsynonymous mutations: Cys35Tyr, Ile155Thr, Pro256Ser (identified in blond/red hair individuals), Vall156Leu and Phe196Leu (in black people) to understand their functional roles on MC1R. First, we estimated their possible phenotypic effects using seven disease-association predictors based on the amino acid sequence of the protein (Table 3, **left**). Only Val156Leu was predicted having neutral effects (5 from 7 predictors), while the remaining were assumed to cause alterations on the MC1R protein that may be linked to a suspected malfunction of the protein.

**Table 3:** Functional and structural impacts of the selected MC1R mutations.

Mutation	Functional Predition							Structural Prediction expressed as ΔΔG (kcal/mol)			
	PolyPhen-2^a^	PON-P2^a^	PANTHER^b^	PhD-SNP^b^	SIFT^b^	SNAP^b^	Meta-SNP^c^	mCSM	SDM	DUET^d^	DynaMut
**C35Y**	***Damaging***	***Pathogenic***	***Disease***	***Disease***	Neutral	***Disease***	***Disease***	***Destabilizing***	***Destabilizing***	***Destabilizing***	Stabilizing
**(rs779504604)**											
	0.998	0.889	0.747	0.876	0.20	0.54	0.693	-0.719	-0.18	-0.651	0.726
**I155T**	***Damaging***	Unknown	***Disease***	***Disease***	***Disease***	***Disease***	***Disease***	***Destabilizing***	***Destabilizing***	***Destabilizing***	***Destabilizing***
**(rs1110400)**											
	0.986	0.781	0.581	0.709	0.03	0.725	0.69	-1289	-1.27	-1178	-0.254
**V156L**	Possibly damaging	Unknown	Neutral	***Disease***	Neutral	***Disease***	Neutral	***Destabilizing***	***Destabilizing***	***Destabilizing***	Stabilizing
**(rs3212365)**											
	0.567	0.519	0.201	0.563	0.23	0.505	0.465	-0.491	-0.99	-0.4987	0.486
**F196L**	***Damaging***	***Pathogenic***	***Disease***	***Disease***	***Disease***	***Disease***	***Disease***	Stabilizing	***Destabilizing***	Stabilizing	Stabilizing
**(rs3212366)**											
	0.997	0.848	0.862	0.837	0.03	0.745	0.734	0.401	-1.64	0.342	0.557
**P256S**	***Damaging***	***Pathogenic***	***Disease***	***Disease***	***Disease***	***Disease***	***Disease***	***Destabilizing***	Stabilizing	***Destabilizing***	***Destabilizing***
**(rs200215218)**											
	1	0.895	0.948	0.907	0	0.765	0.852	-2054	0.53	-1571	-0.569

^a^ PolyPhen-2, PON-P2: Score of probability of the substitution being damaging/pathogenic (normalized value between 0 and 1).

^b^ PANTHER, PhD-SNP, SNAP: Between 0 and 1. (> 0.5 predicted as Disease); SIFT: Positive Value (> 0.05 predicted as Neutral); Consensus Meta-SNP score: Between 0 and 1. (> 0.5 predicted as Disease).

^c^ META-SNP is a meta-predictor that uses scores generated from PANTHER, PhD-SNP, SIFT and SNAP algorithm.

^d^ DUET consensus score.

Next, we decided to further investigate the impact of the amino acid substitutions on the receptor structure. For that, we constructed a three-dimensional (3D) model for MC1R based on the human melanocortin 4 protein receptor (MC4R), with higher sequence identity (>50%) and the muscarinic acetylcholine receptor M2, with higher sequence alignment coverage (0.89). The four structure-based predictions (Table 3, **right**) are centered on changes in ΔΔG energies that result in positive or negative values for stabilizing or destabilizing effects on the receptor structure, respectively. Ile155Thr, Cys35Tyr and Pro256Ser showed agreeable results regarding destabilization of protein structure with the sequence-based predictors associated with damaging effects (Table 3, **left**), suggesting that these three mutations would induce negative effects on MC1R by increasing protein flexibility on the mutation sites.

### The Cys35Tyr mutation could impair the binding of *MC1R* agonists

**Fig 4:**
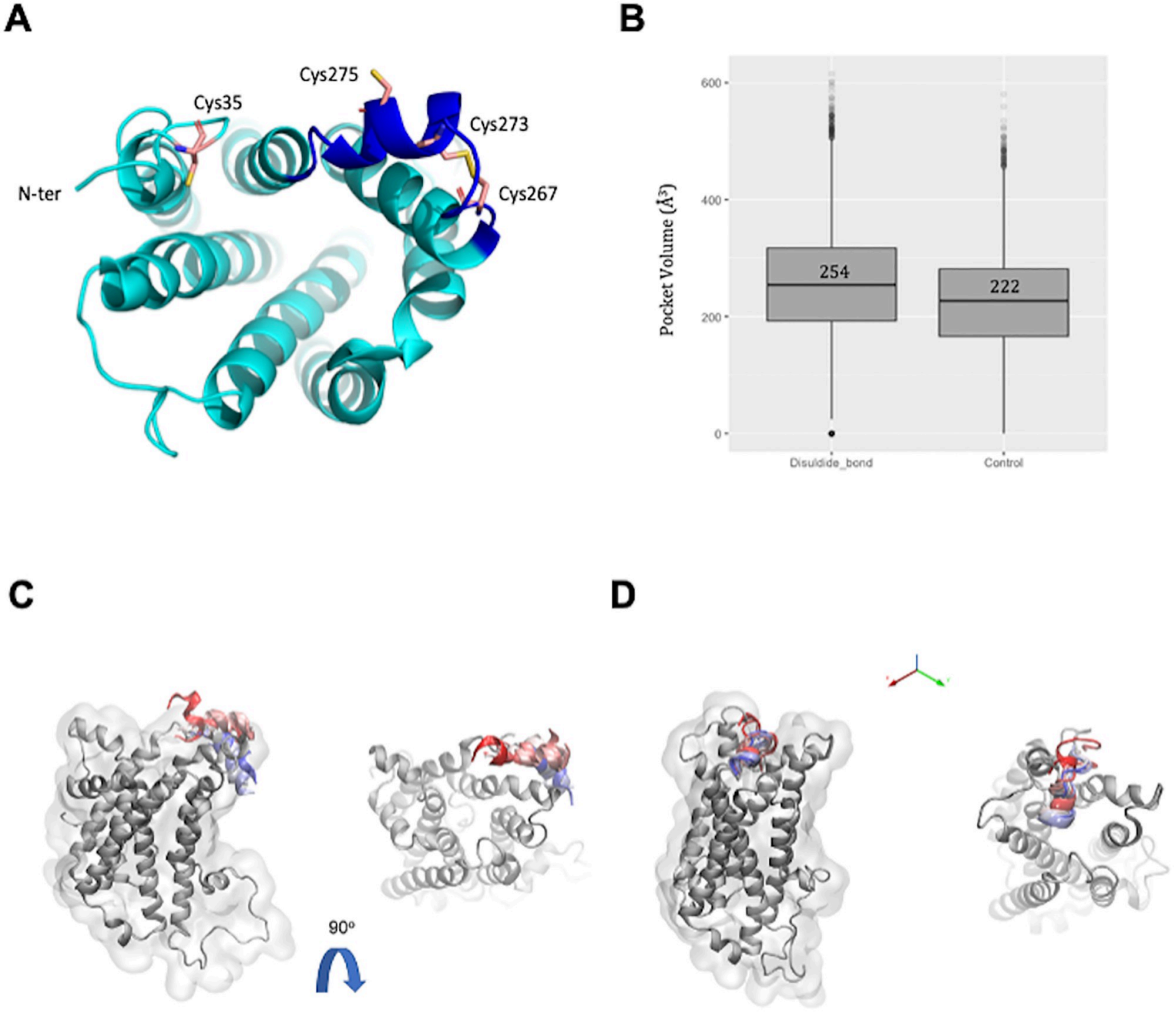
Role of the Cys35-Cys275 disulfide bridge on the *MC1R* structure dynamics. (**A**) *MC1R* structure model is shown as cartoon colored in cyan and four critical cysteine residues are represented as sticks, colored in pink. The extracellular loop 3 is highlighted in dark blue. The disulfide bridge formed by residues 267 and 273 was derived from the MC4R structure used as template for comparative modelling of *MC1R*. Cys35 and Cys275 could be a potential pair for a second disulfide bridge on the protein structure. (**B**) Root Mean Square fluctuation (RMSF) for MC1R residues in presence of the disulfide bond (Cys35-Cys275), in its absence (Cys35) and upon Cys35Tyr mutation. (**C**) *MC1R* binding site volume variation along the MD simulation trajectories. The plot accounts for both MD replicas of *MC1R* model in presence of the disulfide bridge (bridge) and in absence of the disulfide bridge (control). Median values are indicated on the plot. (**D**) *MC1R* conformational changes observed during MD simulations. Representative structures for each system are displayed as a cartoon, colored in cyan. The N-ter first 15 residues are colored in red and the mutation site is indicated as a red sphere. The binding pocket is represented as surface, colored in light orange. When MC1R model bears the Cys35-Cys275 disulfide bond (top), the accessibility of the binding pocket is maintained, with no interference of the N-terminal loop; while in the absence of the bond (middle), the N-terminal loop of MC1R is maintained at the edge of the binding cavity area, interfering with its accessibility. Finally, upon mutation to tyrosine (bottom), the structure suffers an important conformational change that decreases dramatically the pocket volume, and therefore, its capacity to welcome binders.

The nonsynonymous substitution Cys35Tyr was identified in two unrelated red-hair individuals. From the five substitutions selected to be investigated, C35Y presented previous experimental data that indicated a potential role of disrupting a proper function of MC1, directly related to the structure modification. Treatment with reducing agents and induced point mutations on cysteines 35, 267, 273 and 275 have proven that these four cysteine residues are critical [53]. Visual inspection of the 3D structure of MC1R model (Fig 4A) confirmed the two disulfide bond links: Cys267-Cys273 within the extracellular loop 3 (already present on the template MC4R, then transferred to the model) and Cys35-Cys275 between the N-ter and the extracellular loop 3, the latter a potential pair in case the N-terminal loop is in a closer position.

Considering the importance of these post-translational modifications in *MC1R* function, we investigated *MC1R* under three different scenarios:

1. Cys35-Cys275: disulfide bond present;
2. Cys35: disulfide bond absent;
3. Cys35Tyr: disulfide bond absent and mutation on position 35.

Molecular dynamics (MD) simulations were performed for the three MC1R model structures. Root Mean Square Fluctuations (RMSF) is a measure of residue flexibility over the course of the MD simulations. We notice that the absence of the disulfide bond Cys35-Cys275 or the mutation Cys35Tyr increases the overall flexibility of the protein when compared with the structure with the disulfide bond (Fig 4B), making the Cys35Tyr mutant significantly more flexible than the other two systems. This shows that the disulfide bond is important to stabilize the overall protein conformation and the mutation to tyrosine is impacting the overall structure stability.

To further investigate the impact of this increased flexibility on MC1R function, we estimated the average volume of the binding pocket to see if the mutation could impact the binding site region. On the Cys35-Cys275 model, the cavity presents a slightly bigger volume when compared with Cys35, where the disulfide bond is absent (Fig 4C). However, upon mutation to tyrosine, the MC1R binding pocket has a dramatic reduction on the average volume, confirming that the mutation is indeed provoking a conformational change on the receptor that has a significant impact on the integrity of the binding site.

Visual inspection of the MD trajectories and representative structures show that the pocket accessibility is reduced in the absence of the disulfide bond due to the presence of the N-ter loop consistently at the edge of the binding pocket cavity (Fig 4D). Upon mutation to tyrosine, the N-ter loop is not interfering with the binding pocket accessibility, however the increased structure flexibility deforms the overall fold of the receptor and consequently diminishes the binding pocket volume (Fig 4D).

This indicates that Cys35-Cys275 is not only important for the structural integrity of the receptor but also to its function by maintaining the binding pocket accessibility and the Cys35Tyr mutation disrupts the functional conformation of MC1R receptor. In the absence of the disulfide bond, there is an increase of the N-terminal loop hydrogen bonds interactions with the binding pocket edge residues and adjacent loops, while upon mutation there is an increase of the overall receptor flexibility that compromise the receptor fold (S1 Movie). These data suggest that the negative effect associated with the SNP Cys35Tyr might be the rearrangement of the receptor structure disfavoring the access to *MC1R* binders.

## Discussion

This work characterized the entire coding region of melanocortin 1 (*MC1R*) receptor gene in an admixed population from Southeast of Brazil. The *MC1R* is widely studied in humans and non-humans and several variants have been described to influence melanin production [16]. We detected 31 different polymorphisms comprehending synonymous, nonsynonymous substitutions and indels, which agrees with the notion that the *MC1R* is considered a polymorphic gene [54]. The variants associated with damaging effects are commonly genetic alterations that impair the protein function by changing the codon reading frame (indel mutations) or by a single nucleotide substitution resulting in a different codon that produces an amino acid change (nonsynonymous). Based on the knowledge that *MC1R* is better related to red-hair/fair skin phenotype within Europeans or European descendants, in our population all the red hair individuals carry at least one polymorphism known or predicted as loss-of-function variant and, with the exception to Arg151Cys observed in one individual, all the alleles are in heterozygous state. Considering the phase of the alleles is unknown, we cannot affirm whether the *MC1R* mutation in these groups would be sufficient to explain the phenotype.

As mentioned, the *MC1R* gene is highly polymorphic and several altered alleles exhibited low frequency, making any robust correlation analysis between genotype and phenotype difficult. Among the several rare alleles detected in our sample population, three were novel synonymous substitutions: Leu11Leu, Tyr143Tyr and Ala181Ala. Probably, these three substitutions appeared in Brazilian population since the genetic diversity of *MC1R* has been extensively investigated and none of them has been identified in any previous studies or in parental origin populations.

Several theories have been proposed to explain the differences in polymorphic contents on *MC1R* between European and African populations, and the most acceptable is related to ultraviolet radiation b (UVb) exposure [4]. According to Jablonski team, the UV incidence is inversely correlated to latitude; in a constraint pressure towards the maintenance of dark skin in low latitude whereas directional selection is observed in high latitude regions making the skin lightening more advantageous throughout human evolution. A health balance should be achieved in a way to find fine control mechanisms in order to protect the folate from photolysis while at the same time allowing the synthesis of vitamin D. The NMDS based on the genetic diversity of *MC1R* comprising 942bp in length (GRCh37 16:89985667–89986608) was able to efficiently discriminate the three main regions: Africa, Asia and Europe (Fig 2). Although Asians have low levels of melanin on the skin compared to Africans, the lightening of skin occurred as an independent evolutionary process as already demonstrated by other groups [55, 56]. None of the three Brazilian skin color phenotypes clustered to Asians, and this has been supported by the low frequency of Asian matrilineal ancestry (6.7%) in our population. Considering that the significant immigration from Asia to Brazil occurred in the 19^th^ century, it is a recent event in terms of genetic admixture.

As mentioned above, according to the skin reflectance, consequently the Africans are characterized as having dark skin, whereas Europeans are the light ones. The ordination method showed that dark skin group were clustered among Africans explained by the intense traffic of black Africans during the Atlantic slave trade period, especially those from the Bantu ethnic group [57, 58]. Regarding the light skin color, this group gathered with Europeans, in particular Mediterranean populations, in line with the colonization of Rio de Janeiro [20]. Despite the gene flow among Native Americans, Africans and Europeans, the *Fst* value of light and intermediate skin color was very close to that of European populations, indicating similar genetic background in terms of skin color based on *MC1R* (Fig 2 and S3 Table). These findings are not related with mtDNA analysis, in which higher African ancestry was observed in 53% of individuals of intermediate skin, therefore not reflecting the distribution of skin color (Fig 3).

It has already been described that the matrilineal ancestry has an equivalent proportion of European, African and Native American haplogroups among Brazilians, more precisely in Southeastern region [59]. This diversified ancestry profile is present when the total sample population of RJ was analyzed (Fig 3), validating the representativeness of our population of study. However, when the RJ population was categorized based on skin color phenotype, the mtDNA haplogroup distribution was in agreement with the corresponding European and African ancestry origin for light and dark populations, respectively. This is in agreement with the notion that skin color variation in the new world colonies is a product of parental population contribution [12] however, the same conclusion was not reached regarding the intermediate skin color phenotype. Despite the intermediate group being clustered together with light skin populations, the European haplogroup represented only 20% with major contribution from Africans (53%), indicating that the color variation could not be totally explained by matrilineal ancestry inheritance. Analysis using the ancestry-informative markers (AIMs) from somatic chromosomes would be a better strategy for understanding the complexity of color variation in our admixed population.

Based on this AIMs approach, a previous study evaluated several aspects of physical traits in admixed populations from Latin American [23]. Individuals categorized as mixed skin color showed a high percentage of European genetic ancestry (0.69), probably due to the mean melanin index (MI) being more prone to the white spectrum than the black one. It is comprehensible due to the southern region of Brazil having historically received many European immigrants [19, 22] compared to Rio de Janeiro (Southeast), which had higher contribution of African and Native American as demonstrated by another group [20]. However, even considering the colonization particularities between both regions, the intermediate phenotype remains closer to light group as previously observed in GWAS study [23].

Several nonsynonymous polymorphisms present on the coding region of *MC1R* have already been subjected by *in silico* predictors or by *in vitro* assays to evaluate their impacts on receptor function [13, 14]. In this work we have chosen five SNPs; three found in blond-red hair individuals (Cys35Tyr, Ile155Thr and Pro256Ser) and two in black skin individuals (Val156Leu and Phe196Leu). According to the ClinVar database, the SNPs Ile155Thr, Val156Leu and Phe196Leu have been already described as natural variants. ClinVar reports Ile115Thr (VCV000239154.7) as having conflicting interpretations of pathogenicity, Val156Leu (VCV000258651.4) and Phe196Leu (VCV000321433.4) as benign/likely benign, whereas Cys35Tyr (VCV000430181.6) and P256S (VCV000470711.4) showed uncertain significance. Based on these discordant outcomes, we decided to estimate the possible phenotypic effects for all of them using seven disease-association predictors based on the amino acid sequence of the protein. The variants identified in fair skin individuals were predicted to promote negative effect on function as well as destabilize MC1R structure (Table 3). The destabilizing phenotypes are supported by the nature of amino acid substitutions on protein structures. Considering the isoleucine replacement for a threonine could increase polarity and contribute to structure rigidity since its side chain movement is more limited, this could be the reason for the observed phenotype. A meta-analysis on *MC1R* gene showed a significant association between Ile155Thr and the development of melanoma that were corroborated by the fact that this mutation impaired cAMP signaling [60, 61]. Also, Ile155Thr was individually associated with malignant melanoma risk in the Spanish population [62]. Larger, prospective cohorts with different ethnic backgrounds are needed to verify the MC1R variant associations. Regarding Pro256Ser, the proline replacement for a serine on the core of the alpha helix could increase flexibility on this region. Additionally, proline residues in the transmembrane (TM) alpha-helices of integral membrane proteins have long been suspected to play a key role for helix packing and signal transduction by inducing regions of helix distortion and/or dynamic flexibility (hinges), strengthening the idea of Pro256Ser exhibiting a loss-of-function effect. Proteins vary in stability and a ΔΔG in the range of 2 kcal/mol is considered to result in a mutational “hot spot” of sufficient effect [63] on its conformation, required for protein’s function *in vivo*. The predicted changes on protein stability for both mutations have energy values that are inferior or around –2.0 kcal/mol (Table 3), so further studies need to be conducted.

Cys35Tyr was predicted by sequence and structure-based methods as a damaging/destabilizing mutation (Table 3). In addition, previous experimental data have proven that four cysteine residues are critical for the proper function of MC1R, since the treatment with reducing agent DTT as well as induced point mutations on cysteines 35, 267, 273 and 275 impair both ligands interaction with MC1R and cAMP signaling, indicating that disulfide bridges are required for the integrity of the receptor [53].

The role of disulfide bonds has been investigated for the rhodopsin-like family. One disulfide bridge between the extracellular loop 2 and the transmembrane helix 3 is very conserved and increases the constraint on the protein structure [64]. Other disulfide bridges have also been observed, concerning the N-terminal and the extracellular loop 3 for instance on the CXCR4 receptor [65] and within the extracellular loop 3 for human A2A adenosine receptor, melanocortin receptors and human histamine H1 receptor [64], as could be the case for the Cys35 (N-terminal) and Cys275 (extracellular loop 3) on MC1R.

MD simulations of MC1R in absence of the Cys35-Cys275 disulfide bridge have shown that the N-terminal can be flexible and interact with other protein residues, impairing the access to the binding pocket cavity (S1 Movie). Interestingly, MC4R has a tyrosine residue on the same position in sequence for Cys35 at MC1R and the N-terminal portion of the MC1R structure could not be modelled using the MC4R crystal structure as a template, since the equivalent region was missing, which could indicate that it is a very flexible region. We could also assume that an increase of flexibility on this region is expected on MC1R upon mutation.

Our MD simulations of the mutated Cys35Tyr receptor confirmed that hypothesis: tyrosine induces an increase on the overall flexibility of the receptor, leading to a conformational change that dramatically reduces the binding pocket volume. This conformational change could potentially impair the binding of agonists and therefore MC1R function (Fig 4).

Two possibilities could be drawn: a conformational change responsible by impairing agonist binding or the new residue would promote a different signaling, for instance, an antagonist-like signal. The latter hypothesis corroborates previous data on the loss-of-function effect observed *in vitro*, the retention of MC1R in the endoplasmic reticulum [53, 66]. Although the change from cysteine to tyrosine being a natural substitution instead of alanine [66] obtained by mutagenesis, we are not able to confirm whether Cys35Tyr could produce the same outcome, since tyrosine is well known for being phosphorylated and another signaling could be triggered.

In conclusion, we evaluated the genetic diversity of melanocortin-1 receptor (MC1R) in an admixed population from Rio de Janeiro. Analysis based on *MC1R* variants distribution grouped RJ population light and intermediate skin color with Europeans, despite the intermediate matrilineal ancestry being comprised of majority African origin. The gene exhibited a high polymorphic profile among the light skin group with prominent proportion of nonsynonymous substitutions. Among them, several loss-of-function mutations were detected in red-hair individuals. We demonstrated through MD simulations that the Cys35Tyr mutation potentially impairs agonist binding due to the disruption of the disulfide bond Cys35-Cys275 and induced conformational changes on the receptor structure impacting its binding site. This work has shed light on the mutations associated with the *MC1R* gene and contributed to a better understanding of the genetic diversity of *MC1R* in an admixed population with a wide range of color variants.

## Supporting information

S1 Table The 1000 Genomes populations. (XLSX)

S2 Table MAF of *MC1R* polymorphisms from the 1000 Genomes populations. (XLSX)

S3 Table Nei’s pairwise Fst distance for RJ and the 1000 Genomes populations. Pairwise FSTs among populations from the 1000 Genome Consortium and Rio de Janeiro. The Fst is given for each pair of populations in the lower left triangle of the matrix. The upper right triangle of the matrix is shown the confidence interval (lower limit—upper limit).
(XLSX)

S4 Table Nei’s pairwise Fst distance of phenotypes of hair and eye color. (XLSX)

S5 Table mtDNA haplotypes (freq). (XLSX)

S1 Movie *MC1R* model structures upon the three different studied scenarios: With the Cys35-Cys275 disulfide bond (left); in the absence of the disulfide bond (middle); and upon the Cys35Tyr mutation (right). (MP4)
